# Photocatalytic degradation of toxic aquatic pollutants by novel magnetic 3D-TiO_2_@HPGA nanocomposite

**DOI:** 10.1038/s41598-018-33818-9

**Published:** 2018-10-19

**Authors:** Chella Santhosh, A. Malathi, Ehsan Daneshvar, Pratap Kollu, Amit Bhatnagar

**Affiliations:** 1 Department of Environmental and Biological Sciences, University of Eastern Finland, P.O. Box 1627, FI-70211 Kuopio, Finland; 2 Department of Electronics and Communication Engineering, KLEF, Greenfields, Vaddeswaram, Vijayawada, 522502 India; 3 Solar Energy Lab, Department of Chemistry, Thiruvalluvar University, Vellore, 632115 India; 4 CASEST, School of Physics, University of Hyderabad, Gachibowli, Hyderabad 500046 India; 5 Newton Alumnus fellow, Thin film magnetism group, Cavendish Laboratory, University of Cambridge, Cambridge, CB3 0HE UK

## Abstract

In this study, a series of photocatalysts were prepared, namely bare 3D-TiO_2_ (*b-3D-T*), magnetic 3D-TiO_2_: (*m3D-T*) and magnetic 3D-TiO_2_@Hierarchical Porous Graphene Aerogels (HPGA) nanocomposite: (*m3D-T-HPGA NC*) by solvothermal process. The prepared photocatalysts were analyzed by using X-ray diffraction (XRD), Field emission scanning electron microscope (FE-SEM), transmission electron microscope (TEM), Vibrating sample magnetometer (VSM), Brunauer–Emmett–Teller (BET) and Diffuse Reflectance Measurement – Ultraviolet (DRS-UV) to know their physical and chemical properties. The photocatalytic degradations of two toxic aquatic pollutants viz., Cr(VI) and bisphenol A (BPA) were tested by using the prepared photocatalysts. Parameters such as initial pollutant concentration, solution pH, photocatalyst dosage, wavelength and light intensity were investigated to optimize the process. The photocatalytic properties of prepared catalyst were analyzed based on the degradation of Cr(VI) and BPA under UV irradiation. The modified photocatalysts showed better performance as compared to b-3D-T photocatalyst. This better performance is ascribed to efficient charge transfer between b-3D-T nanoparticles to the porous graphene sheets. The maximum photocatalytic degradation of Cr(VI) was found to be 100% with m3D-T-HPGA NC within 140 min, whereas a removal efficacy of 100% and 57% was noticed in case of m3D-T and b-3D-T within 200 and 240 min, respectively. In the case of BPA, the maximum degradation efficiency was found to be 90% with m3D-T-HPGA NC within 240 min.

## Introduction

Water pollution, due to increased agricultural and industrial activities, has badly affected the quality of life and eco-system in the past few decades^1^. The effective removal of various toxic pollutants from the industrial wastewater remains a big challenge to date. The major toxic inorganic pollutants include metal ions, whereas dyes, phenols and polychlorinated biphenyls are some of the organic pollutants present in water and wastewater^2,3^. Among different potential toxic elements, chromium (Cr) is one of the main inorganic pollutants. Chromium mainly exists in two oxidation states, Cr(III) and Cr(VI). Compared to Cr(III), Cr(VI) is 100 times more toxic in nature and carcinogenic^4,5^. Hence, there is a need to remove or reduce toxic Cr(VI) to less toxic Cr(III), which can then be precipitated as Cr(OH)_3_ under alkaline conditions^6^. On the other hand, bisphenol A (BPA) is one of the toxic emerging pollutants, which is most commonly used as a raw material for producing epoxy resins, polycarbonate plastic, flame retardants and some other chemical products^7–9^. Based on its huge production and use for different industrial applications, BPA has been discharged into aquatic environment without proper treatment from the industries^10,11^. Direct discharge of BPA to the environment and fresh water bodies is harmful to the human health and aquatic life. Hence, the removal of organic and inorganic pollutants from wastewater is necessary before discharging them to the environment.

Various technologies have been proposed and studied to remove the toxic pollutants from water and wastewater, which include ion exchange, adsorption process, membrane process, advanced oxidation process, and photocatalysis. Among these technologies photocatalysis is considered as a green technology for water treatment because the mechanism relies on the light irradiation to convert toxic pollutants to non-toxic^12^. Basically, the mechanism of photocatalysis is to convert the photon energy into the chemical energy^13^. Here, the photocatalyst plays an important role that generates a transient state by using light absorption (photon energy) and releasing the electron-hole pair to produce the chemicals (chemical energy) in the form of products^13^.

Among different catalysts, semiconductor photocatalysts have shown significant efficiency towards the degradation of organic and inorganic pollutants. TiO_2_ is one of the commercial and efficient photocatalyst material used for the degradation of many pollutants, due to its versatility, ease to synthesize and good controllability and stability^14,15^. However, there are some disadvantages of using TiO_2_ as catalyst, as it gets activated only in UV region, which uses <5% of the solar light. Based on these drawbacks, many researchers have focused on the preparation of different structures of TiO_2_ and their nanocomposites which helps to degrade the toxic pollutants in water and wastewater^16,17^. The performance of TiO_2_ depends on the high surface area, different sizes and shape and surface modification^18^. However, there is a problem of recovering the material after photocatalysis process. Thus, to avoid the material separation problems, magnetic photocatalyst could be prepared which can easily be recovered from the aqueous solution by applying the external magnetic field^19^. To increase the photocatalytic performance of the catalyst and charge transfer characteristics, doping or modification with high surface area materials is also needed.

Graphene is a carbon family member with 2D structure and high surface to volume ratio which is used in diverse applications. Recently, compared with 2D graphene sheets, 3D graphene sheets have gained much attention due to their high porosity, high surface to volume ratio, low mass density and high electrical conductivity^20^. Hierarchical porous graphene aerogels (HPGA) have shown good efficiency compared with 2D graphene sheets^21^. The porous networks of the graphene sheets could be capable of more ion and mass transport to the semiconductor materials. Based on this idea, a nanocomposite with TiO_2_ and HPGA is synthesized in the present study, where HPGA is expected to facilitate the charge, mass and ion transfer by involving 3D-TiO_2_ and cobalt ferrites (CoFe_2_O_4_), hence this could act as an efficient photocatalyst for the degradation of toxic pollutants.

The present work deals with the preparation and characterization of a series of photocatalysts namely bare 3D-TiO_2_ (*b-3D-T*), magnetic 3D-TiO_2_: (*m3D-T*) and magnetic 3D-TiO_2_@Hierarchical Porous Graphene Aerogels (HPGA) nanocomposite: (*m3D-T-HPGA NC*). The structural and crystalline properties of the prepared nanocomposites were characterized by using FE-SEM, TEM, XRD, VSM, BET and DRS-UV techniques. The photocatalytic degradation of Cr(VI) and BPA was examined to evaluate the photocatalytic performance of prepared photocatalysts. Among the three prepared materials, m3D-T-HPGA NC shows the highest degradation of the selected pollutants and acts as efficient photocatalyst with good magnetic property for easy recovery of the material from the water.

## Results and Discussion

### Characterization of photocatalysts

**Figure 1:**
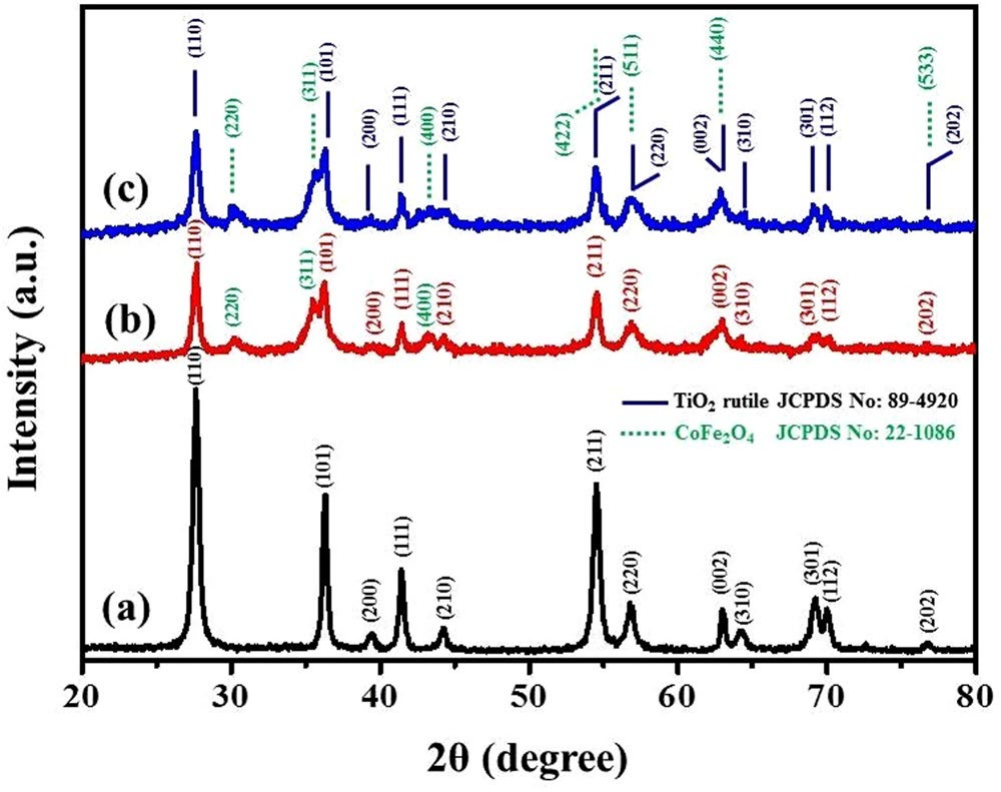
XRD analysis of (**a**) bare 3D-TiO_2_, (**b**) magnetic 3D-TiO_2_, (**c**) magnetic 3D TiO_2_@HPGA nanocomposite.

XRD analysis was done to know the crystal structures of prepared nanocomposites viz., b-3D-T, m3D-T and m3D-T-HPGA NC. Figure 1(a) shows the XRD analysis of b-3D-T, which exhibited a dominant rutile phase with diffraction peaks of 27.5°, 36.3°, 39.4°, 41.5°, 44.1°, 54.5°, 56.8°, 62.9°, 64.3°, 69.2°, 70.0° and 76.69° with a corresponding crystal planes of (110), (101), (200), (111), (210), (211), (220), (002), (310), (112) and (202), respectively. All the peaks are in good agreement with the standard JCPDS No: 21-1276^13,22^. Figure 1(b) shows the XRD analysis of m3D-T nanocomposite. The diffraction peaks at 30.39°, 35.43°, 43.3°, 54.5°, 56.9°, 63.0° and 76.6° were observed which indexed to the crystal planes of (220), (311), (400), (422), (511), (440) and (533), respectively. All the diffraction peaks are ascribed to the spinel-type cobalt ferrite (CoFe_2_O_4_), which is in good agreement with the standard JCPDS No: 22-1086^23^. Fig. S1(a) shows the XRD analysis of bare CoFe_2_O_4_ nanoparticles which is in good agreement with the above crystal planes and diffraction peaks. Figure 1(c) shows the XRD analysis of m3D-T-HPGA NC. All the diffraction peaks and crystal planes are matched with the m3D-T nanocomposite. No diffraction peak of hierarchal porous graphene aerogel (HPGA) was observed in the prepared nanocomposite. Fig. S1(b) shows (002) and (101) crystal plane at 2θ of 26.7° and 41.2° diffraction peaks respectively, which confirms the presence of graphene hydrogel peak. The presence of (002) and (101) was disappeared in the nanocomposite (Fig. 1(c)) because graphene sheets were decorated on the surface of the b-3D-T and CoFe_2_O_4_ nanoparticles and the detection of thin graphene layers was difficult^24^. Hence, the prepared nanocomposites were synthesized without any impurities by using the solvothermal process.

### Morphological analysis

**Figure 2:**
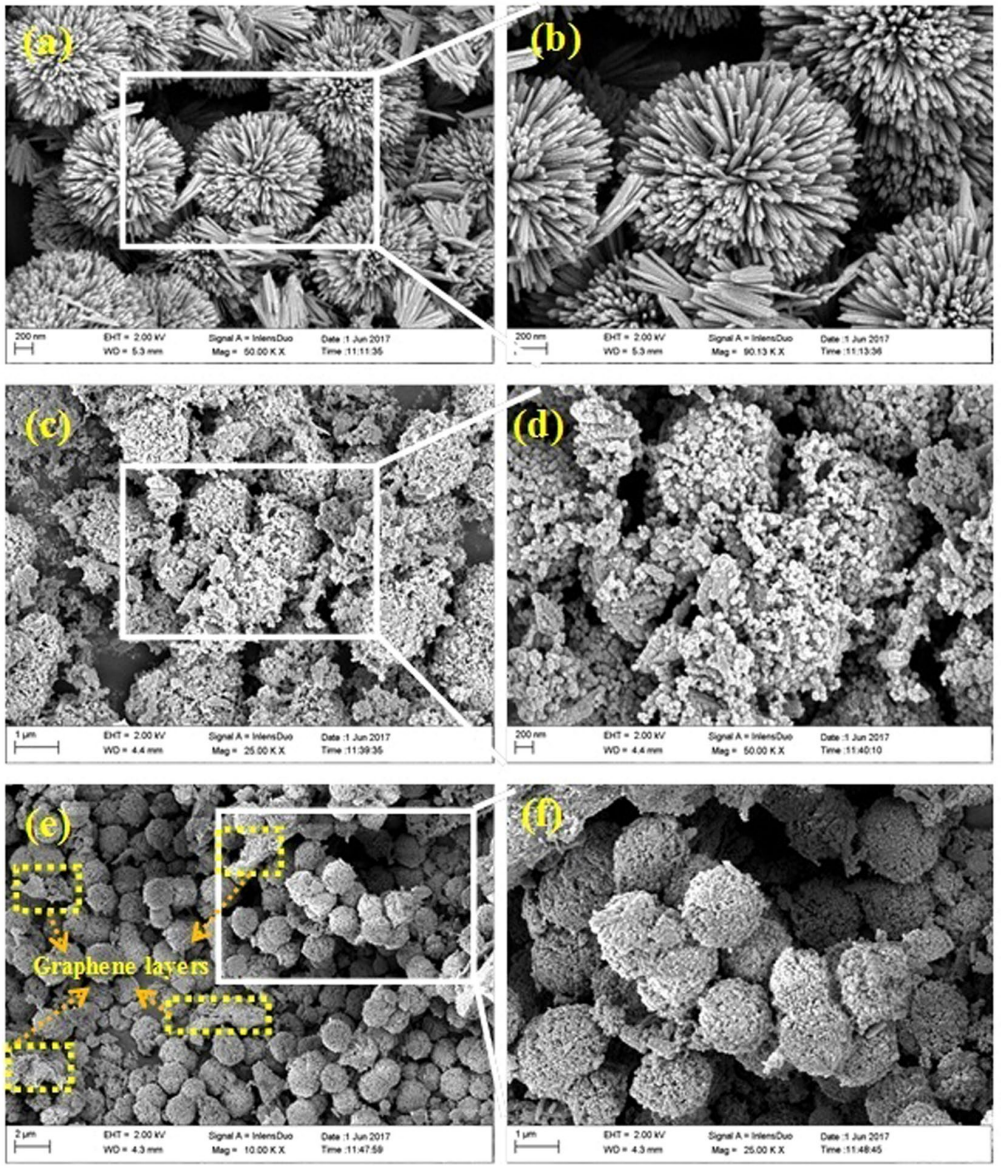
FE-SEM images of (**a**,**b**) flower like 3D-TiO_2_, (**c**,**d**) magnetic 3D-TiO_2_ and (**e**,**f)** magnetic 3D-TiO_2_@HPGA at different magnifications.

Figure 2(a) shows the FE-SEM images of b-3D-T material synthesized in this study. Flower like structures of TiO_2_ were seen from the images (Fig. 2(a)). Each TiO_2_ flower like structures consists of nanorods in hierarchical form possessing a microsphere. Figure 2(b) shows a zoom-in view of flower like 3D-TiO_2_ microspheres. Each individual sphere possesses an average size of 1.5 µm, whereas each nanorod was found to have an average diameter of 20–30 nm with a length of 300–350 nm. As seen from the images, each individual nanorod was self-assembled to form a 3D-TiO_2_ microsphere with a high compact hierarchical structure. With such a well confined structure, it could enhance the photocatalytic properties by facilitating the charge carriers transport easily. Figure 2(c) shows the SEM image of m3D-T nanocomposite, which reveals that as compared to b-3D-T, the magnetic particles (CoFe_2_O_4_) were well anchored on the surface of 3D-TiO_2_. The average particle size of the magnetic nanoparticles was in the diameter of 20–25 nm. As clearly seen from the images (Fig. 2(d)), the tips of 3D-TiO_2_ nanorods were covered fully by the CoFe_2_O_4_ nanoparticles. Hence the prepared nanocomposite was successfully synthesized by the solvothermal process. Finally, Fig. 2(e,f) shows the m3D-T-HPGA NC. As compared to the m3D-T, CoFe_2_O_4_ nanoparticles were well decorated without any agglomeration. This might be explained due to the incorporation of HPGA on to the m3D-T, and as a result, graphene layers help to increase the surface to volume ratio as well as avoiding the agglomeration between the magnetic nanoparticles.

**Figure 3:**
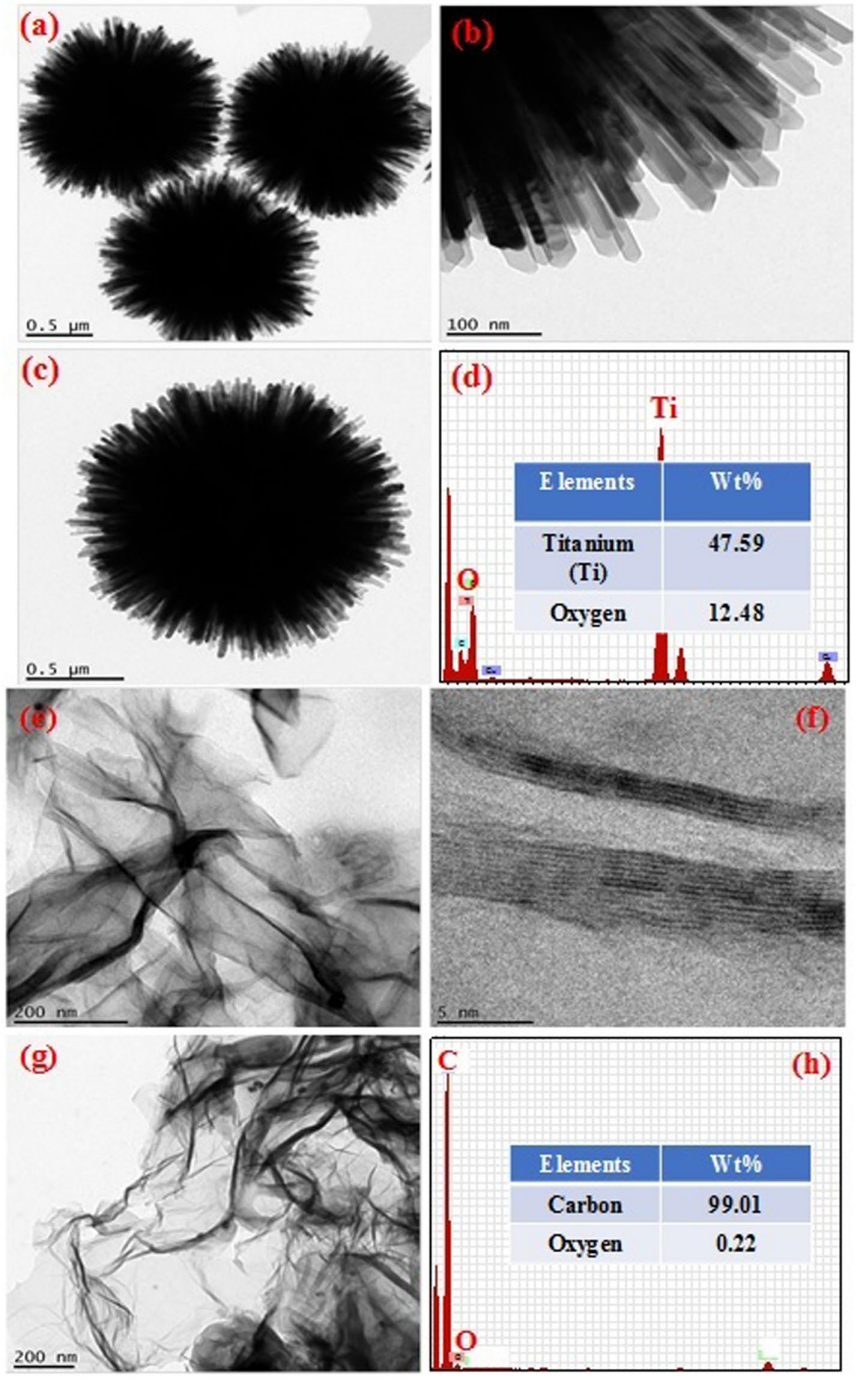
TEM images of (**a**–**d**) bare 3D-TiO_2_ and **(e**–**h**) bare HPGA with EDAX analysis.

Figure 3 shows the TEM images with EDAX of b-3D-T and bare HPGA nanomaterials. Figure 3(a–d) clearly shows the flower like 3D-TiO_2_ spheres with EDAX analysis. From the figures, it can be clearly seen that a bunch of nanorods (1D) were formed to a flower like structure (3D). The diameter of each nanorod was approximately in the range of 20–25 nm size. The nanorod looks like 1D structures (Fig. 3b), and these bunch of nanorods then form 3D flower like TiO_2_ structures (Fig. 3(a,c)). Figure 3d shows the EDAX analysis of b-3D-T, which reveals the elements present in the material. The major element present in the b-3D-T nanomaterial is titanium (Ti) (47.59 in wt.%) and oxygen (O) (12.48 wt.%). Some other elements are also present in the sample such as copper, which arises from the TEM copper grids. Figure 3(e–h) clearly shows the graphene flakes with few number of sheets with a d-spacing of 0.347 nm. Figure 3(h) shows the EDAX of HPGA and the major elements present in the material are carbon (C) and oxygen (O). The carbon is in the highest amount (99.01 wt.%), whereas oxygen is 0.22 wt.% and the remaining is copper which arises from TEM copper grids.

Figure S2 shows the TEM images with EDAX of m3D-T and m3D-T- HPGA NC. Figure S2(a–d) clearly shows that the flower like 3D-TiO_2_ spheres were fully decorated by the magnetic (CoFe_2_O_4_) nanoparticles. From the figures, it can be clearly seen that all the 3D flower like TiO_2_ spheres were well decorated with the magnetic nanoparticles. Fig. S2(d) shows the EDAX analysis of m3D-T, which reveals the elements present in the material. The major elements present in the m3D-T nanomaterial are oxygen (O) (35.89 in wt.%), titanium (Ti) (24.4 in wt.%), iron (Fe) (32.08 in wt.%) and cobalt (Co) (7.64 in wt.%). Some other elements are also present in the sample such as copper, which arises from the TEM copper grids. Fig. S2(e–h) shows the m3D-T-HPGA NC with EDAX analysis, and it can be seen from the figures that the m3D-T nanomaterial was decorated on graphene flakes. Fig. S2(h) shows the EDAX of m3D-T-HPGA NC and the major elements present in the material are carbon (C) (10.74 in wt.%), oxygen (O) (25.79 in wt.%), titanium (Ti) (24.4 in wt.%), iron (Fe) (32.03 in wt.%) and cobalt (Co) (7.04 in wt.%). Further ICP-MS analysis was also performed with the prepared materials to know the real composition. Both EDAX and ICP-MS analyses showed the same weight percentage of the prepared materials. The values of the ICP-MS analysis are shown in Table 1.

**Table 1:** ICP-MS analysis of bare 3D-TiO_2_, magnetic 3D-TiO_2_ and magnetic 3D-TiO_2_@HPGA.

Prepared materials	Ti (g/kg)	O (g/kg)	Co (g/kg)	Fe (g/kg)	C (g/kg)
Bare 3D-TiO_2_	618	382	—	—	—
Magnetic3D-TiO_2_	284	368	114	234	—
Magnetic 3D-TiO_2_@HPGA	292	298	110	190	110

### VSM analysis

**Figure 4:**
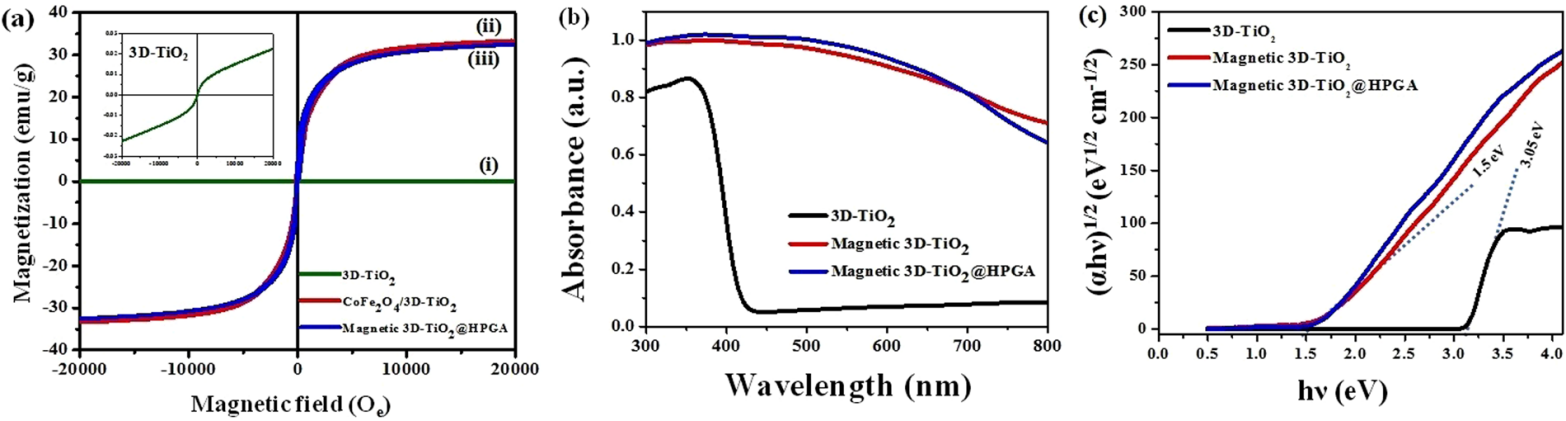
(**a**) M-H hysteresis of prepared nanocomposites (i) bare 3D-TiO_2_, (ii) magnetic 3D-TiO_2_ and (iii) magnetic 3D-TiO_2_@HPGA (Inset figure shows the extended form of bare 3D-TiO_2_), (**b**) Diffuse reflectance (DRS) UV-vis spectrum and (**c**) band gap calculations of synthesized materials.

Figure 4(a) shows the magnetic hysteresis (M-H) of prepared nanocomposites viz. b-3D-T, m3D-T and m3D-T-HPGA NC. Figure 4a(i) shows the M-H hysteresis of b-3D-T spheres, which is almost zero because TiO_2_ is a non-magnetic material. Inset figure also shows that the saturation magnetization value of TiO_2_ is 0.02 emu/g, which is negligible and equals to zero. Figure 4a(ii) shows the M-H hysteresis of m3D-T nanocomposite. This material shows 33 emu/g of saturation magnetization, which resembles that the material is well coated with the CoFe_2_O_4_ nanoparticles onto the 3D-TiO_2_ spheres. Magnetic 3D-TiO_2_@HPGA (m3D-T-HPGA NC) also shows the good magnetic property with a saturation magnetization of 32 emu/g (Fig. 4a(iii)), (note that HPGA is also a non-magnetic material). Hence, there is slight difference between the m3D-T and m3D-T-HPGA NC. Overall, the hysteresis loop analysis suggests that the material is well decorated with the magnetic nanoparticles (CoFe_2_O_4_) onto the 3D-TiO_2_ and HPGA nanomaterials with a super paramagnetic in nature. This is very important for recovering and reuse of the material from water and wastewater after the process.

### Optical properties

The diffuse reflectance UV-visible spectrum of b-3D-T, m3D-T and m3D-T-HPGA NC are shown in Fig. 4(b). The prepared b-3D-T exhibits the higher absorbance in UV region and the maximum absorption peak arises at about 354 nm, whereas in the case of m3D-T and m3D-T-HPGA NC, there was a broad peak in the region of 300 to 800 nm. The broad peak was observed due to the blue shift of the absorption edges of m3D-T and m3D-T-HPGA NC as compared to b-3D-T. Using Kubelka-Munk function^25^, the band gap energy values were calculated for the prepared nanocomposites, which are shown in Fig. 4(c). The plot shows the intercept line towards the x-axis, which gives the bandgap energy value of the prepared nanocomposites. The b-3D-T photocatalyst shows the bandgap energy of 3.05 eV and whereas m3D-T and m3D-T-HPGA NCs show the bandgap energy of 1.5 eV. The bandgap energy is low for the two nanocomposites as compared to b-3D-T material, which may be due to the introduction of magnetic nanoparticles and HPGA materials to the TiO_2_ material. The change in size and shape might have caused a change in the band gap of the material. The addition of magnetic particles will lead to easy recovery of the nanomaterial from the water, whereas the role of HPGA is to increase the surface area and it will have more recombination rates, which will finally result in the degradation of the pollutants within the specified time.

**Figure 5:**
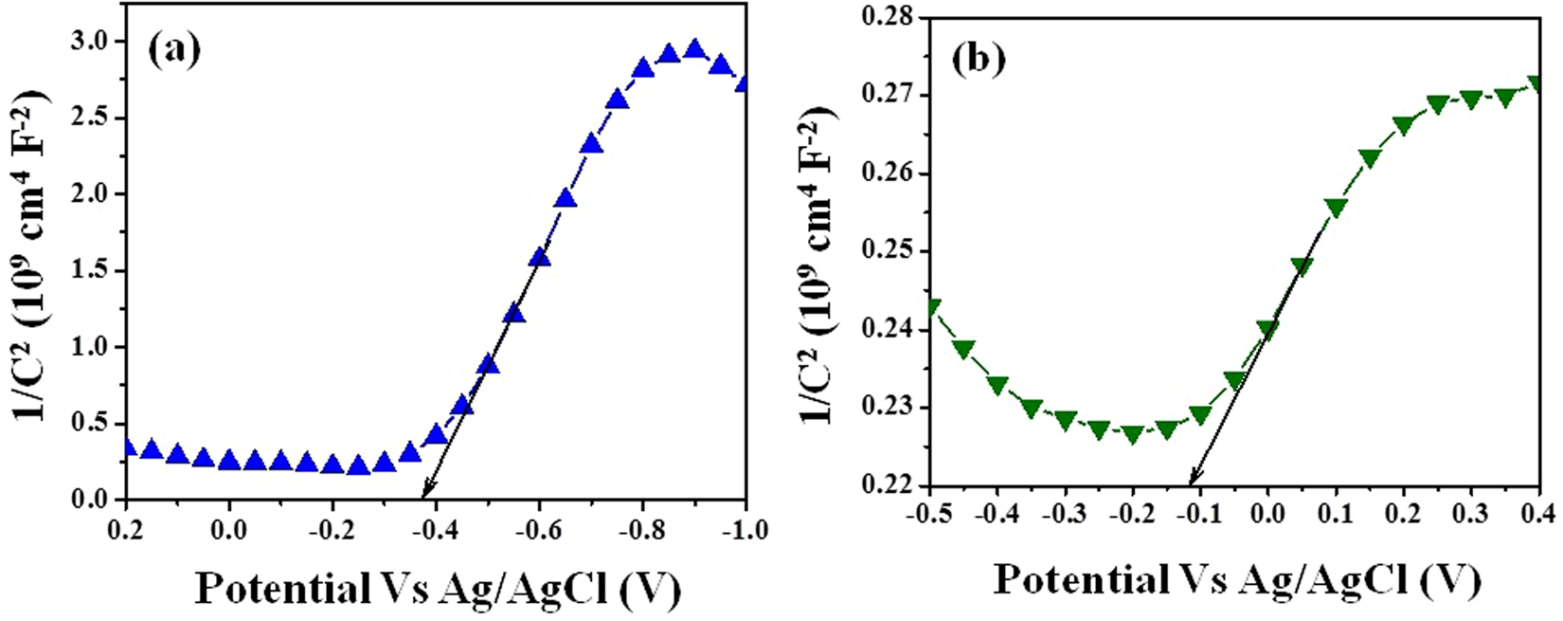
Mott-Schottky plots of (**a**) magnetic 3D-TiO_2_ and **(b**) magnetic 3D-TiO_2_@HPGA nanocomposite.

Figure 5 shows the Mott-Schottky plots for the m3D-T and m3D-T-HPGA NC. As shown in Fig. 5(a,b), the potentials of conduction band (CB) for m3D-T and m3D-T-HPGA NC are −0.34 eV and −0.15 eV, respectively. Thus, the valence band (VB) potentials of m3D-T and m3D-T-HPGA NC are calculated to be +1.16 eV and +1.35 eV, based on the CB value and the band gap (1.5 eV) obtained from the optical absorption spectrum.

### BET surface area and pore size analysis

The textural properties of prepared materials were characterized by BET analysis. The N_2_ adsorption/desorption analysis of the magnetic 3D-TiO_2_@HPGA is shown in Fig. S3a. The specific surface area of b-3D-T, m3D-T and m3D-T-HPGA NC were found to be 21.23, 31.63 and 45.97 m^2^/g, respectively. As compared to the bare materials, the composite (m3D-T-HPGA NC) shows a higher surface area, which is due to hierarchical porous graphene aerogel (HPGA) with meso-pore structures. By using Barrett-Joyner-Halenda (BJH) model, the pore distribution of the prepared nanocomposites was analyzed and shown in Fig. S3b. The prepared m3D-T-HPGA showed that the average pore size distribution is 14.25 nm.

### Photoelectrochemical studies

**Figure 6:**
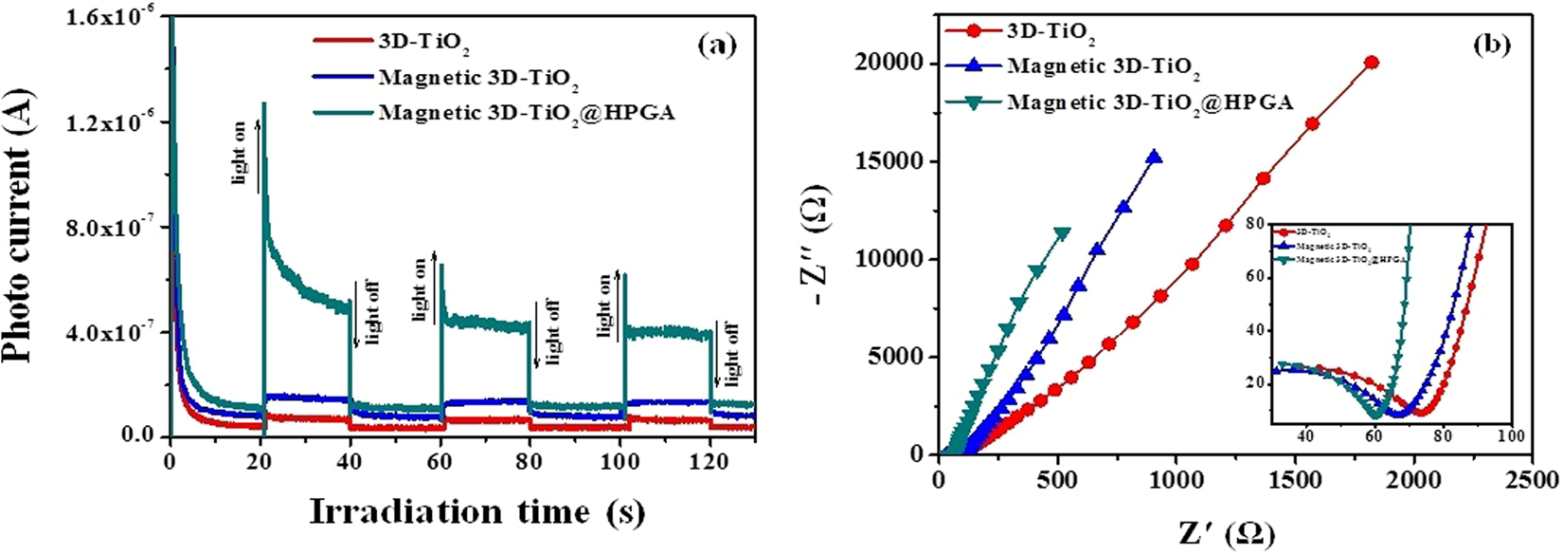
(**a**) Transient photocurrent response and (**b**) Nyquist plot for 3D-TiO_2_, magnetic 3D-TiO_2_ and magnetic 3D-TiO_2_@HPGA nanocomposite (Inset figure shows the extended form of Nyquist plot).

The photocurrent and electrochemical impedance spectroscopy (EIS) are vital characterization techniques to investigate the separation and migration of charge carriers in the photocatalysts. Figure 6 shows the photocurrent response of b-3D-T, m3D-T and m3D-T-HPGA NC with three on-off cycles under UV - irradiation. From the photocurrent results (Fig. 6(a)), it can be observed that m3D-T-HPGA NC exhibited a higher photocurrent intensity as compared to m3D-T and b-3D-T, which reveals that the charge separation efficiency in m3D-T-HPGA NC was higher than other catalysts and hence, it can generate more photo induced charge carriers. In addition, EIS measurement was performed to investigate the charge transfer resistance of the material. Figure 6(b) shows the Nyquist plot of b-3D-T, m3D-T and m3D-T-HPGA NC, respectively. As seen from Fig. 6(b), m3D-T-HPGA NC possesses a smaller arc radius than the m3D-T and b-3D-T. The smaller arc radius of m3D-T-HPGA NC implies the lower at the contact interface. This further proves that the interfacial charge-transfer and separation efficiency of photo excited charge carriers is higher for m3D-T-HPGA NC which is much valuable for its superior photocatalytic activity.

### Photocatalytic degradation studies

#### Effect of photocatalyst dosage

**Figure 7:**
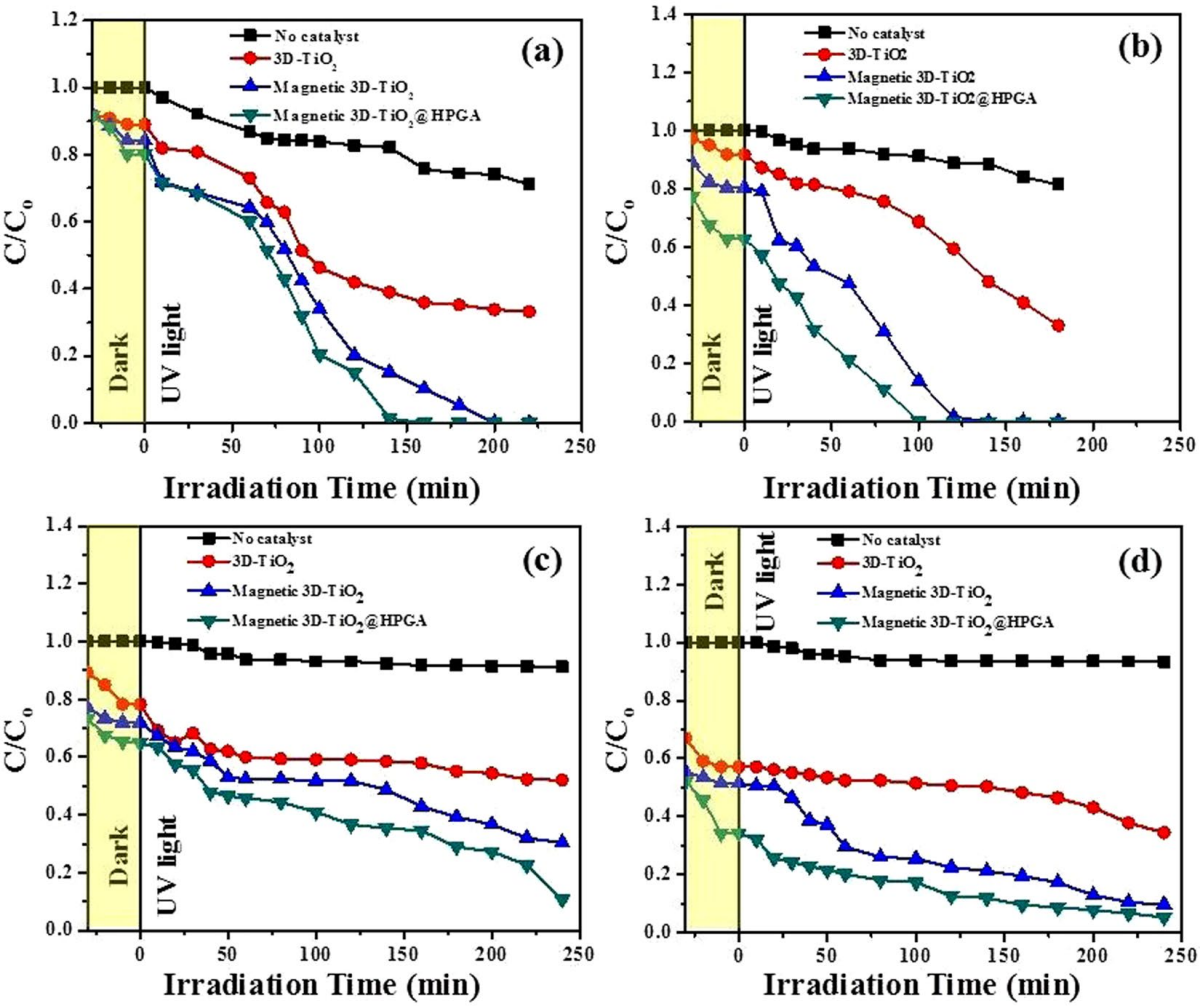
Effect of photocatalyst dosage on Cr(VI) and BPA degradation under UV irradiation. (**a**,**b**) 0.1 and 0.2 g L^−1^ of photocatalysts for the degradation of 10 mg L^−1^ of Cr(VI) at pH 2 under 254 nm, (**c**,**d**) 0.1 and 0.2 g L^−1^ of photocatalysts for the degradation of 10 mg L^−1^ of BPA at pH 5.64 under 365 nm.

Figure 7(a,b) shows the effect of photocatalyst dosage on the degradation of Cr(VI) with respect to the degradation ratio (C/C_o_) and various intervals of time (t). Figure 7(a) shows the photodegradation of 10 mg L^−1^ Cr(VI) ions with a catalyst dosage of 0.1 g L^−1^ under pH 2 with a wavelength of 254 nm and with 4 lamps intensity (32 W). Cr(VI) concentration was found to reduce to some extent in the absence of photocatalyst. This suggests that UV light has some minor effect on the degradation of Cr(VI). However, in the presence of b-3D-T, degradation of Cr(VI) was observed (50–60% in 240 min). Furthermore, the photocatalytic activity of m3D-T shows a significant degradation of Cr(VI) ions, and within 200 min 100% degradation of Cr(VI) was achieved. Moreover, in the case of m3D-T-HPGA NC, a 100% degradation of Cr(VI) ions was achieved within 140 min. The mechanism of photocatalytic activity is schematically represented in Fig. S4 and briefly explained as follows. When UV light is illuminated on the photocatalyst (3D-TiO_2_), electron hole pair is generated, where the electrons are excited at its conduction band and holes in its valency band. The reduction of Cr(VI) to Cr(III) subsequently occurs due to photoexcited electrons (e^−^), and water molecule is oxidized to O_2_, due to the holes (h^+^) generated in the valency band. The degradation efficiency was found to be two times higher for m3D-T-HPGA NC than b-3D-T, which can be explained due to the fact that m3D-T-HPGA possesses high specific surface area and enhanced generation of electron-hole pairs. Figure 7(b) shows the photodegradation of 10 mg L^−1^ Cr(VI) ions with a catalyst dosage of 0.2 g L^−1^ under similar conditions. Among the three photocatalysts, m3D-T-HPGA NC shows the best results and 100% degradation of Cr(VI) ions was achieved within 100 min under UV irradiation. Fig. S5(a) shows that the prepared nanocomposites followed the pseudo first order kinetic model for Cr(VI) ions^26^.

Figure 7(c,d) shows the effect of catalyst dosage on the degradation of BPA with respect to the degradation ratio (C/C_o_) and at various intervals of time (t). Figure 7(c) shows the photodegradation of 10 mg L^−1^ BPA with a catalyst dosage of 0.1 g L^−1^ under pH 5.64 with a wavelength of 365 nm and with 4 lamps intensity (32 W). A negligible decrease in BPA concentration was observed in the absence of photocatalyst. This suggests that UV light alone does not have any major effect on the degradation of BPA. However, in the presence of b-3D-T and m3D-T, degradation of BPA took place, but only 20 and 40% of BPA was degraded in 240 min. Furthermore, in the case of m3D-T-HPGA NC, 70–80% degradation of BPA occurred within 240 min. Figure 7(d) shows the photodegradation of 10 mg L^−1^ BPA with a catalyst dosage of 0.2 g L^−1^ under similar conditions. Among the three photocatalysts, m3D-T-HPGA NC showed the best results and almost 90% degradation of BPA was achieved within 240 min under UV irradiation. Fig. S5(b) shows that the prepared nanocomposites followed the pseudo first order kinetic model for BPA.

#### Effect of initial concentration of Cr(VI) and BPA

**Figure 8:**
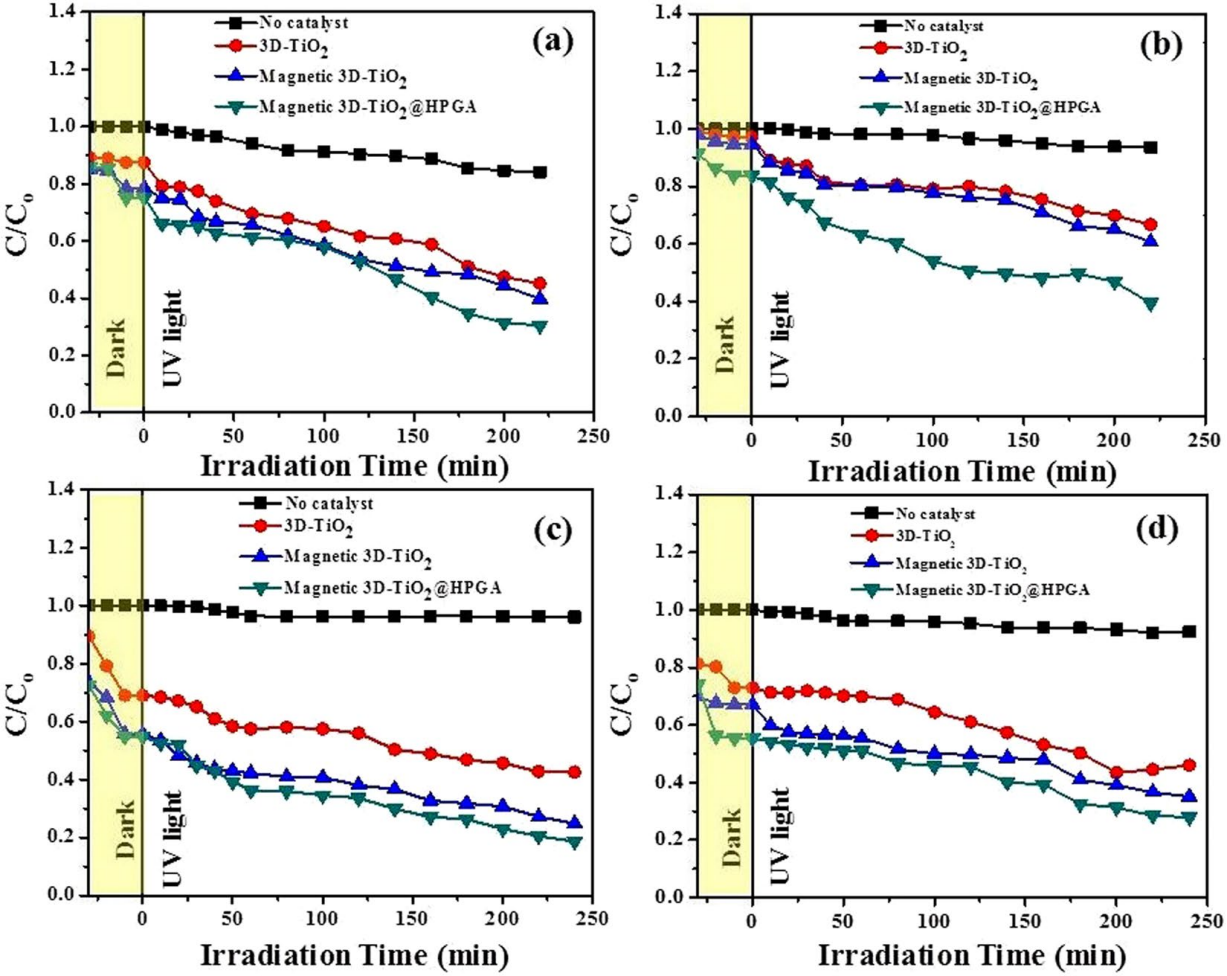
Effect of initial concentration of Cr(VI) and BPA degradation under UV irradiation. (**a**,**b**) 25 and 50 mg L^−1^ of Cr(VI) with 0.1 g L^−1^ of photocatalysts at pH 2 under 254 nm, (**c**,**d**) 25 and 50 mg L^−1^ of BPA with 0.1 g L^−1^ of photocatalysts at pH 5.64 under 365 nm.

Figure 8 (a,b) shows the effect of initial concentration of studied pollutants on the degradation of Cr(VI) ions with respect to the degradation ratio (C/C_o_) and various intervals of time (t). Figure 8(a) shows the photodegradation of 25 mg L^−1^ Cr(VI) ions with a catalyst dosage of 0.1 g L^−1^ under pH 2 with a wavelength of 254 nm and with 4 lamps intensity (32 W). Figure 8(b) shows the photodegradation of 50 mg L^−1^ Cr(VI) ions under similar conditions. The concentration of Cr(VI) ions remains same in the absence of photocatalyst. As the initial Cr(VI) concentration increases, there is a decrease in the degradation of Cr(VI) ions from 60 to 45% in 220 min for 25 and 50 mg L^−1^ of Cr(VI) respectively. This is because there are less electron hole pairs as the concentration of Cr(VI) ions is high. So, there will be a decrease in the degradation process. Figure 8(c,d) shows the effect of initial concentration on the degradation of BPA with respect to the degradation ratio (C/C_o_) and at various intervals of time (t). Figure 8(c) shows the photodegradation of 25 mg L^−1^ BPA with a catalyst dosage of 0.1 g L^−1^ under pH 5.64 with a wavelength of 365 nm and with 4 lamps intensity (32 W). Figure 8(d) shows the photodegradation of 50 mg L^−1^ BPA under similar conditions. The degradation percentage of BPA was decreased from 70 to 55%, due to the high concentration of BPA. If the concentration of pollutant is high, the generation of electron and hole pairs will not be enough to degrade the pollutant. Due to this reason, low degradation of BPA occurs at higher BPA concentrations.

#### Effect of solution pH

**Figure 9:**
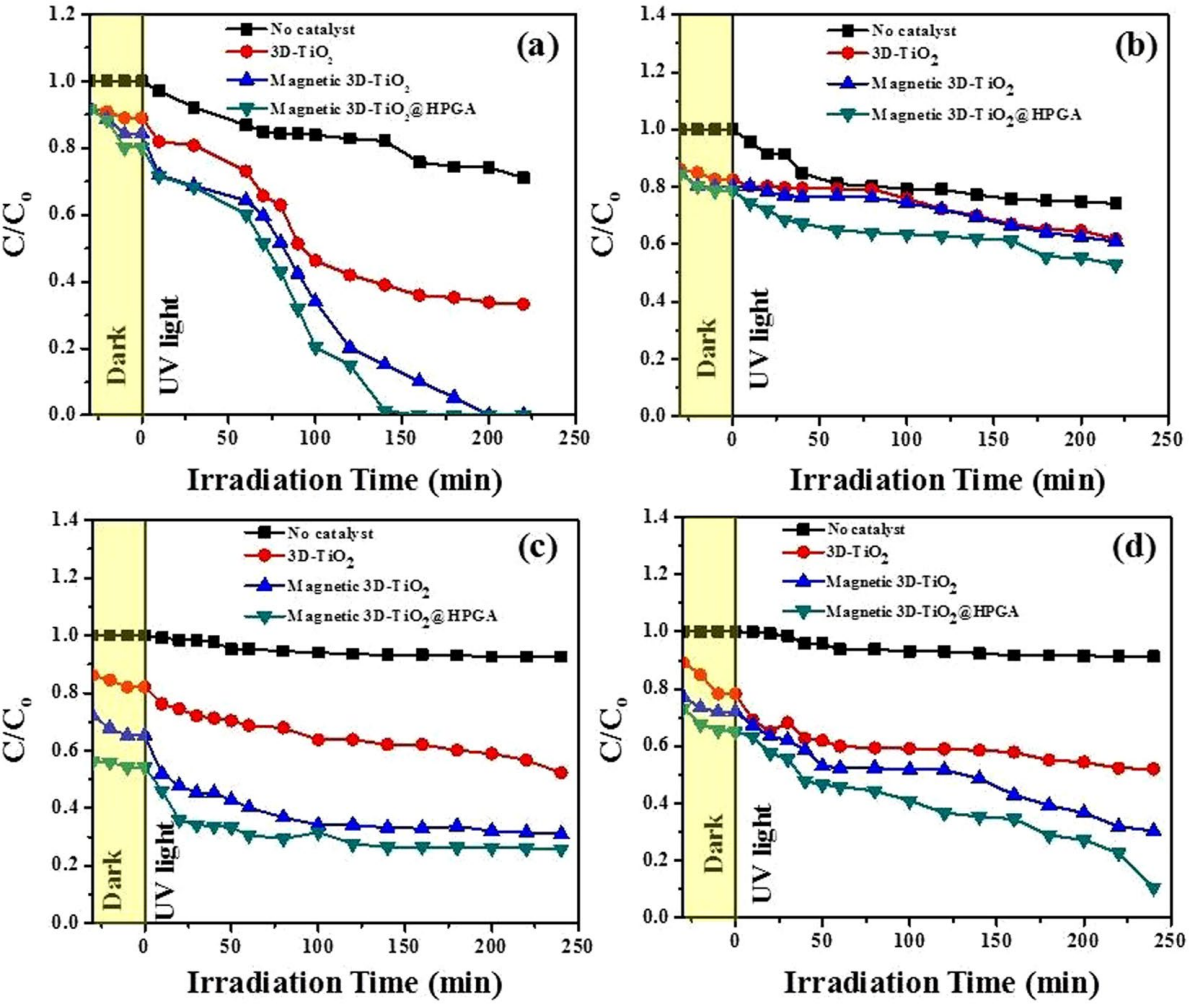
Effect of pH on Cr(VI) and BPA degradation under UV irradiation. (**a**,**b**) pH 2 and 5.64 of Cr(VI) with 0.1 g L^−1^ of photocatalysts and 10 mg L^−1^ of Cr(VI) under 254 nm, (**c**,**d**) pH 2 and 5.64 of BPA with 0.1 g L^−1^ of photocatalysts and 10 mg L^−1^ of BPA under 365 nm.

Figure 9(a,b) shows the effect of solution pH on the degradation of Cr(VI) with respect to the degradation ratio (C/C_o_) and various intervals of time (t). Figure 9(a) shows the photodegradation of 10 mg L^−1^ Cr(VI) ions with a photocatalyst dosage of 0.1 g L^−1^ at pH 2 with a wavelength of 254 nm and with 4 lamps intensity (32 W). Figure 9(b) shows the photodegradation of 10 mg L^−1^ Cr(VI) at pH 5.6 under similar conditions. As seen from Fig. 9(a,b), Cr(VI) was well degraded in acidic pH than normal pH (5.64). At pH 2, Cr(VI) ions degraded almost 100% within 140 min. Whereas, Cr(VI) ions were degraded approximately 20 to 30% of the initial concentration under normal pH (5.64) (Fig. 9(b)). It reveals that in acidic pH, more negatively charged Cr(VI) species in the form of CrO_4_^2−^ were associated with the positive charged surface of photocatalyst via electrostatic attraction which leads to the substantial degradation of Cr(VI) ions in acidic pH^27^. As the pH increases from 2 to 5.64, there is a decrease in the degradation of Cr(VI) ions, which is due to the electrostatic repulsion of negative charged surface and negative charged Cr(VI) species on the photocatalyst surface. Hence, pH 2 was selected for the degradation of Cr(VI) for the remaining parameters. Figure 9(c,d) shows the effect of pH on the degradation of BPA with respect to the degradation ratio (C/C_o_) and at various intervals of time (t). Figure 9(c) shows the photodegradation of 10 mg L^−1^ BPA with a photocatalyst dosage of 0.1 g L^−1^ under pH 2 with a wavelength of 365 nm and with 4 lamps intensity (32 W). Figure 9(d) shows the photodegradation of 10 mg L^−1^ BPA under pH 5.64 with similar conditions. After observing Fig. 9(c,d), BPA is well degraded at normal pH than acidic pH. At pH 2, degradation of BPA is less, because at acidic pH, the surface of the photocatalyst and BPA were positively charged, hence, there was repulsive force between them. Therefore, little degradation occurred in acidic pH. In the case of normal pH (5.64), the degradation of BPA is quite good and it degraded upto 90% in 240 min, where the positive charge of BPA and negative charge of catalyst favors the degradation^28^. Hence, normal pH was chosen for the degradation of BPA for the remaining parameters.

#### Effect of UV wavelength

**Figure 10:**
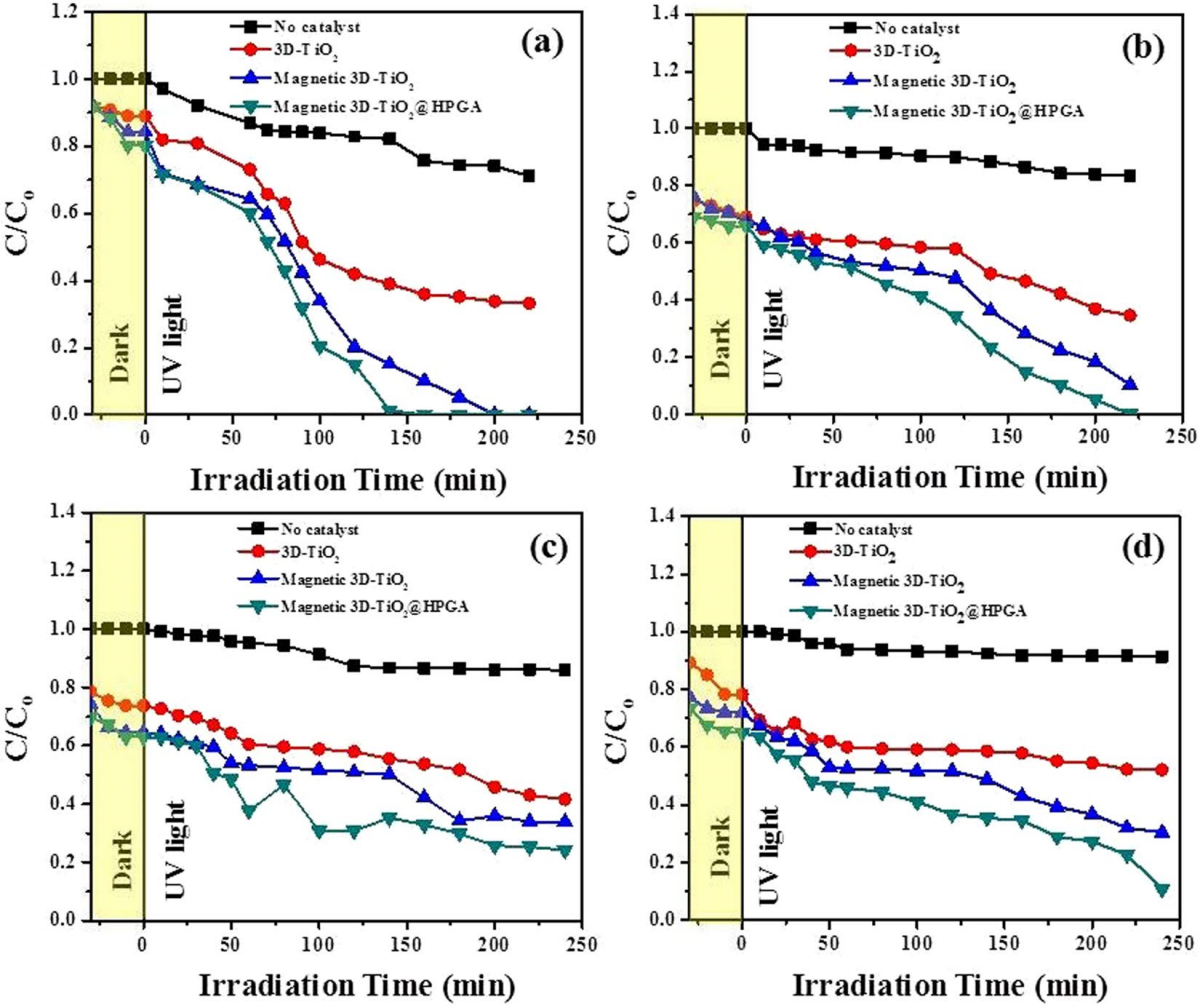
Effect of wavelength on Cr(VI) and BPA degradation under UV irradiation. (**a**,**b**) 254 and 365 nm for Cr(VI) degradation with 0.1 g L^−1^ of photocatalysts and 10 mg L^−1^ of Cr(VI) at pH 2, (**c**,**d**) 254 and 365 nm for BPA degradation with 0.1 g L^−1^ of photocatalysts and 10 mg L^−1^ of BPA at pH 5.64.

Figure 10(a,b) shows the effect of UV wavelength on the degradation of Cr(VI) ions with respect to the degradation ratio (C/C_o_) and various intervals of time (t). Figure 10(a) shows the photodegradation of 10 mg L^−1^ Cr(VI) ions with a photocatalyst dosage of 0.1 g L^−1^ under pH 2 with a wavelength of 254 nm and with 4 lamps intensity (32 W). Figure 10(b) shows the photodegradation of 10 mg L^−1^ Cr(VI) ions under the wavelength of 365 nm under similar conditions. It was found that both UV wavelengths (254 and 365 nm) have considerable effect on the degradation of Cr(VI) ions, but in the case of 254 nm, Cr(VI) ions were more effectively degraded in a short period of time, as compared to 365 nm. Hence, for all the parameters, 254 nm wavelength was chosen for degradation of Cr(VI) ions. Figure 10(c,d) shows the effect of UV wavelength on the degradation of BPA with respect to the degradation ratio (C/C_o_) and various intervals of time (t). Figure 10(c) shows the photodegradation of 10 mg L^−1^ BPA with a photocatalyst dosage of 0.1 g L^−1^ under pH 5.64 with a wavelength of 254 nm and with 4 lamps intensity (32 W). Figure 10(d) shows the photodegradation of 10 mg L^−1^ BPA under the wavelength of 365 nm under similar conditions. It was found that both UV wavelengths (254 and 365 nm) have considerable effect on degradation of BPA, but in the case of 365 nm, BPA was more effectively degraded as compared to 254 nm. Hence, for all the parameters, 365 nm was chosen for the degradation of BPA.

#### Effect of light intensity

**Figure 11:**
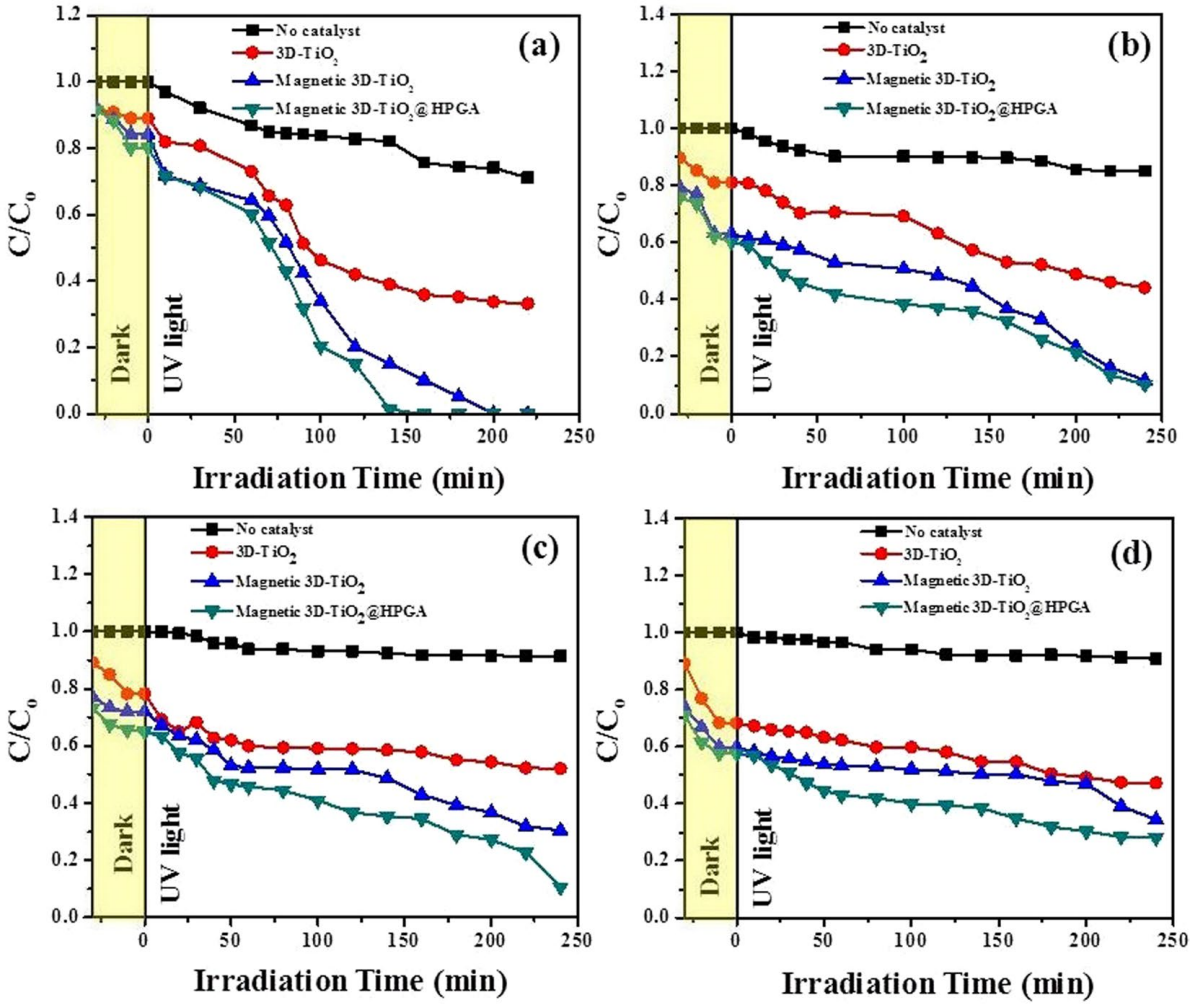
Effect of light intensity on Cr(VI) and BPA degradation under UV irradiation. (**a**,**b**) 32 and 16 W of light intensity for Cr(VI) degradation with 0.1 g L^−1^ of photocatalysts and 10 mg L^−1^ of Cr(VI) at pH 2 under 254 nm, (**c,d**) 32 and 16 W of light intensity for BPA degradation with 0.1 g L^−1^ of photocatalysts and 10 mg L^−1^ of BPA at pH 5.64 under 365 nm.

Figure 11(a,b) shows the effect of light intensity on the degradation of Cr(VI) ions with respect to the degradation ratio (C/C_o_) and various intervals of time (t). Figure 11(a) shows the photodegradation of 10 mg L^−1^ Cr(VI) ions with a photocatalyst dosage of 0.1 g L^−1^ under pH 2 with wavelength of 254 nm and with 4 lamps light intensity (32 W). Figure 11(b) shows the photodegradation of 10 mg L^−1^ Cr(VI) ions under 2 lamps light intensity (16 W) under similar conditions. Both the intensities were found effective for the degradation of Cr(VI) ions with different degradation times. Cr(VI) ions were degraded within 140 min with 32 W light intensity (Fig. 11(a)), but with 16 W light intensity, degradation of Cr(VI) was slower (Fig. 11(b)). Hence, for the all parameters, 32 W light intensity was chosen for Cr(VI) ions degradation. Figure 11(c,d) shows the effect of light intensity on the degradation of BPA with respect to the degradation ratio (C/C_o_) and various intervals of time (t). Figure 11(c) shows the photodegradation of 10 mg L^−1^ BPA with a photocatalyst dosage of 0.1 g L^−1^ under pH 5.64 with a wavelength of 365 nm and with 4 lamps intensities (32 W). Figure 11(d) shows the photodegradation of 10 mg L^−1^ BPA under 2 lamps light intensities (16 W) with similar conditions. It was found that both the intensities helped in degradation of BPA also with degradation times. BPA was degraded 90% within 240 min with 32 W light intensity (Fig. 11(c)), but with 16 W light intensity, degradation of BPA was slower (Fig. 11(d)). Hence, for the all parameters, 32 W light intensity was chosen for BPA degradation. Table 2 shows the comparison between different photocatalysts with the prepared photocatalysts (in this study), which reveals that the photocatalysts (prepared in this study) show the better performance in the degradation of Cr(VI); however, in case of BPA, a comparable removal efficiency was found^26,29–34^.

**Table 2:** Comparison of different photocatalyst materials (from literature) with the prepared photocatalyst materials (this study).

Pollutant	S. No	Photocatalyst	Operational parameters					References
			Degradation Time (min)	pH	Catalyst dosage (g L^−1^)	Initial concentration of pollutants (mg L^−1^)	Degradation (%)	
Cr(VI)	1.	ZnO	15	6	12	300	50	^26^
	2.	ZnO	120	4.5	12	100	50	^29^
	3.	TiO_2_	300	2	1	10	70	^30^
	4.	TiO_2_	180	2.5	1	150	80	^31^
	5.	m3D-T-HPGA	140	2	0.1	10	100	***This study***
Bisphenol A (BPA)	6.	SnO_2_	60	—	—	25	94.3	^32^
	7.	TiO_2_	60	—	—	25	98.1	^32^
	8.	ZnO	60	—	—	25	99.9	^32^
	9.	TiO_2_	60	7	0.5	20	>90	^33^
	10.	Mpg-C_3_N_4_	360	3.05–11	0.5	20	100	^34^
	11.	m3D-T-HPGA	240	5.6	0.1	10	90	***This study***

The absorbance spectra of Cr(VI) and BPA under UV-irradiation are shown in Fig. S6(i & ii). Cr(VI) degradation was monitored by the time-evolution of absorbance spectra. Fig. S6(i) shows the typical absorbance spectra of Cr(VI) solution with irradiation time with all the three photocatalysts. Among all the three photocatalysts, magnetic 3D-TiO_2_@HPGA NC showed the better degradation efficiency within 140 min. As seen from the figure, the absorbing intensity of Cr(VI) decreases with irradiation time, indicating the rapid photodegradation of Cr(VI). The same was also noticed for the degradation of BPA (Fig. S6(ii)).

### Effect of scavengers and TOC analysis

**Figure 12:**
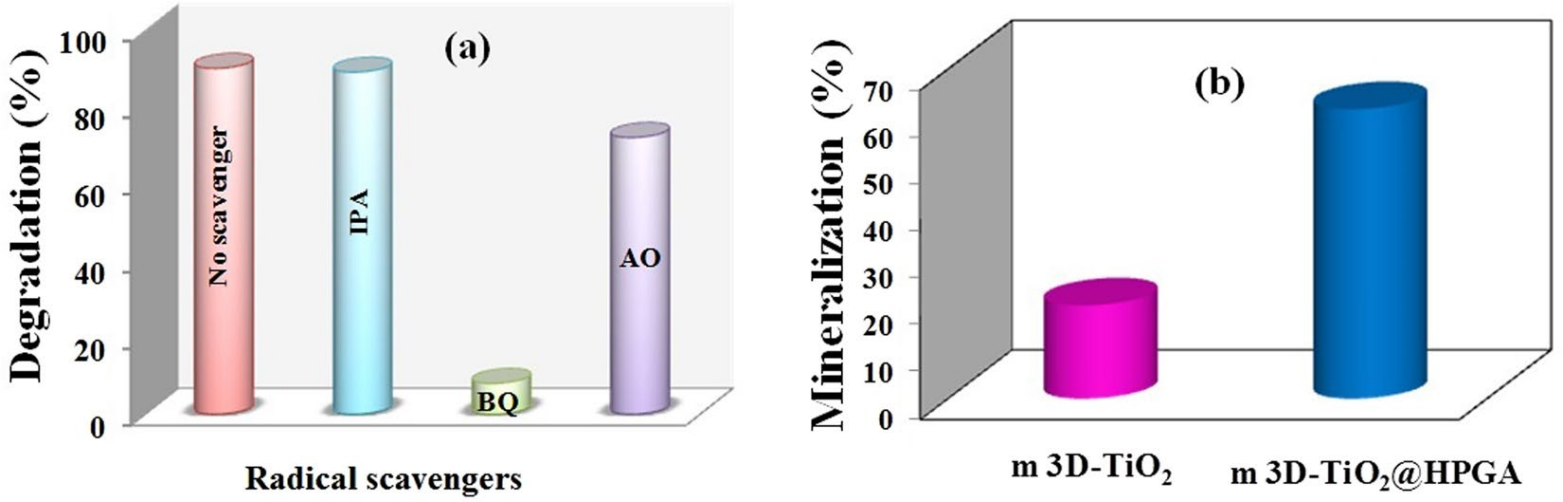
(**a**) Effect of scavengers on the photocatalytic degradation of BPA over m3D-T-HPGA NC under UV – irradiation and (**b**) Mineralization of BPA with photocatalytic degradation using magnetic 3D-TiO_2_ and magnetic 3D-TiO_2_@HPGA nanocomposite.

In photodegradation experiment, the pollutants are oxidized by some reactive species such as holes (h^+^), superoxide radicals (O_2_^•−^) and hydroxyl radicals (^•^OH). The radical trapping experiment was performed to identify the reactive species involved in the photodegradation of BPA by m3D-T-HPGA NC with various kinds of radical scavengers such as ammonium oxalate (AO), benzoquinone (BQ) and isopropyl alcohol (IPA) and the obtained results are depicted in Fig. 12(a). In this experiment, IPA (1 mM) was added to scavenge ^•^OH and 89% degradation was observed when compared to no scavengers (90%). This revealed that ^•^OH did not involve in the degradation reaction. Furthermore, the addition of BQ (1 mM) and AO (1 mM) scavenged O_2_^•−^ and h^+^. About 8% and 72% of BPA degradation was observed by BQ and AO, respectively, when compared with no scavenger. The results revealed that O_2_^•−^ and h^+^ are the important reactive species responsible for the degradation of BPA. Further, it could be concluded from the results that O_2_^•−^ and h^+^ are the major and minor species for the photocatalytic degradation of BPA by m3D-T-HPGA NC.

In addition, total organic carbon (TOC) analysis was performed to investigate the mineralization of photo-degraded BPA solution using m3D-T and m3D-T-HPGA NC. The mineralization of BPA was calculated to be 24 and 67% respectively on m-3D-T and m3D-T-HPGA NC. (Fig. 12b). A 4-fold enhancement was achieved using m3D-T-HPGA NC, clearly indicating the efficiency of m3D-T-HPGA NC for mineralization of BPA solution.

### Photostability and reusability studies

**Figure 13:**
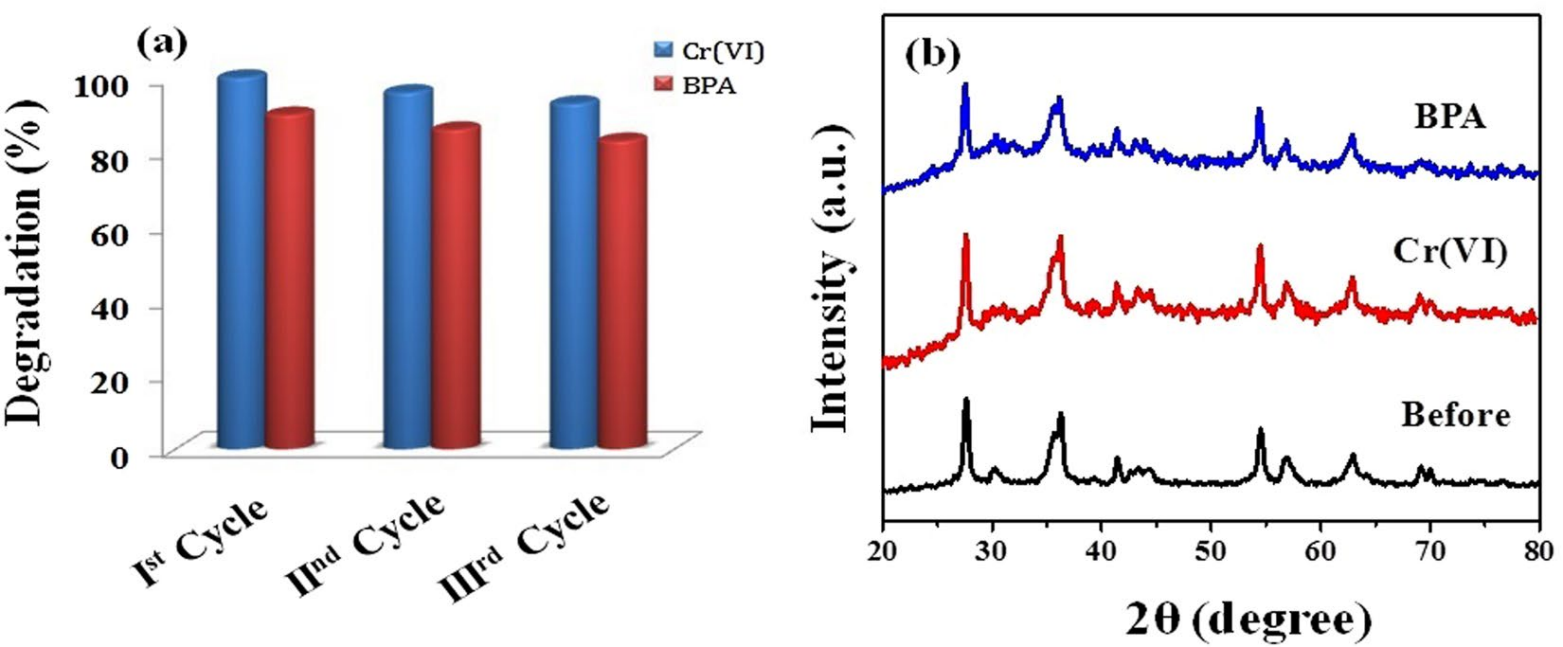
(**a**) Recycling test run for the catalytic decomposition of Cr(VI) and BPA under illumination over m3D-T-HPGA NC and (**b**) its corresponding XRD spectra of m3D-T-HPGA NC before and after recycling test of the catalytic degradation of Cr(VI) and BPA.

The photostability and reusability of photocatalyst are of vital importance for its commercial applications. The photostability and reusability features of m3D-T-HPGA NC were evaluated by the photocatalytic degradation of Cr(VI) and BPA under illumination for three cyclic runs. In every cycle, the recovered material was washed, centrifuged and annealed at 60 °C for 6 h. Then the recovered material was weighed again to add the lost amount and used for the next cycle. As shown in Fig. 13(a), the photodegradation efficiency of m3D-T-HPGA NC for the degradation of Cr(VI) and BPA almost remained same even after three cycles, which shows that the prepared m3D-T-HPGA NC is reusable. Figure 13(b) depicts the XRD patterns of fresh and used m3D-T-HPGA NC. From the results, it can be seen that the peak positions and crystal structure of used m3D-T-HPGA NC are similar to the fresh m3D-T-HPGA NC. This inferred that m3D-T-HPGA NC is stable under degradation experiment and it can be used over a long period of time.

## Conclusions

Three photocatalyst materials, viz. b-3D-T, m3D-T and m3D-T-HPGA NCs were successfully synthesized by both hydrothermal and solvothermal processes. The prepared nanocomposite (m3D-T-HPGA NCs) showed 100% and 90% degradation of Cr(VI) and BPA, respectively under UV illumination. Present study reports the performance of prepared nanocomposites by varying different parameters such as, photocatalyst dosage, initial pollutant concentration, solution pH, light wavelength and light intensity. Compared to b-3D-T and m3D-T nanocomposites, m3D-T-HPGA shows the better degradation over Cr(VI) and BPA pollutants. At pH 2 and 5.64, Cr(VI) and BPA pollutants were degraded well with the wavelength of 254 (Cr(VI)) and 365 (BPA) nm and with 32 W light intensity. Overall, the prepared photocatalyst nanocomposite shows the better performance towards the reduction of Cr(VI) and BPA. Decorating magnetic particles onto the 3D-TiO_2_ is an advantage which is useful for easy recovery of material from the treated water.

## Materials and Methods

### Materials

Analytical grade chemicals were used in this study without any purification. Tetrabutyl titanate ((TBT), Sigma Aldrich), titanium tetrachloride (Alfa Aesar), toluene (99.9%, Sigma Aldrich), ethylene glycol (VWR Chemicals), ethanol, iron(III) chloride hexahydrate (Sigma Aldrich), cobalt chloride hexahydrate (Acros Organics), polyethylene glycol (PEG-8000, Alfa Aesar), sodium acetate (Alfa Aesar), graphite powder (Fisher Chemicals), sulphuric acid (98%, VWR Chemicals), sodium nitrate (Sigma Aldrich), potassium permanganate (Sigma Aldrich), hydrogen peroxide (30%, Alfa Aesar), cobalt oxide (50–80 nm diameter, Alfa Aesar), potassium dichromate (Sigma Aldrich), bisphenol A (Sigma Aldrich) were used in this study.

### Synthesis of 3D-TiO_2_

The 3D-TiO_2_ nanoparticles were synthesized by using hydrothermal method^13^. In brief, titanium tetrachloride (TiCl_4_) was added drop-wise to the deionized water in an ice bath with vigorous stirring to obtain 50 wt.% of TiCl_4_ aqueous solution. Simultaneously, 4 ml of tetrabutyl titanate (TBT) was added drop wise to 30 ml of toluene in an ice bath under vigorous stirring. Subsequently, 50 wt.% obtained TiCl_4_ solution was added drop-wise to the TBT solution under stirring for 1 h. After 1 h, the obtained white precipitate was then transferred to the stainless-steel Teflon autoclave and kept for 24 h at 150 °C. After 24 h, the obtained material was centrifuged and washed with DI water and ethanol for several times. Finally, the material was kept for drying in vacuum oven at 50 °C and denoted as bare 3D-TiO_2_ (b-3D-T).

### Synthesis of magnetic 3D-TiO_2_

Magnetic 3D-TiO_2_ nanocomposite was prepared by using solvothermal process. In brief, 50 ml of ethylene glycol containing 2 mmol of iron chloride and 1 mmol of cobalt chloride was kept for ultrasonication for 30 min. Then, 0.2 g of 3D-TiO_2_ nanoparticles were added to the iron-cobalt solution under magnetic stirring for 30 min. Later, 3.6 g of sodium acetate and 1.0 g of polyethylene glycol were added to the mixture and stirred for 30 more min. The mixture was then transferred to stainless-steel Teflon autoclave and kept at 200 °C for 12 h. Finally, the mixture was centrifuged, washed several times with DI water and ethanol, and then kept for drying at 50 °C, and denoted as magnetic 3D-TiO_2_ (3D-TiO_2_@CoFe_2_O_4_) (m3D-T).

### Synthesis of magnetic 3D-TiO_2_@HPGA nanocomposite

Graphene oxide (GO) was synthesized by modified Hummer’s method. Hierarchical porous graphene aerogel (HPGA) was prepared by the method reported in the literature^21^ using GO as precursor material. The magnetic 3D-TiO_2_@HPGA nanocomposite was prepared by using solvothermal process. In brief, 50 ml of ethylene glycol containing 0.5 g of HPGA, 2 mmol of iron chloride and 1 mmol of cobalt chloride was kept for ultrasonication for 1 h. Then 0.2 g of 3D-TiO_2_ nanoparticles were added to the iron/cobalt solution under magnetic stirring for 30 min. Later, 3.6 g of sodium acetate and 1.0 g of polyethylene glycol were added to the mixture and stirred for 30 more min. Then the mixture was transferred to stainless-steel Teflon autoclave and kept at 200 °C for 12 h. Finally, the mixture was centrifuged, and washed several times with DI water and ethanol, and then kept for drying at 50 °C, and denoted as magnetic 3D-TiO_2_@HPGA (m3D-T-HPGA NC).

### Photocatalytic studies

Heber compact multi wavelength and multilamp photo reactor (Model: HML-COMPACT-LP-MP88) was used in this study for the degradation of Cr(VI) and bisphenol A (BPA). The photocatalytic experiments were done under UV light irradiation. Photoreactor consists of 4 sets of 8 W low pressure mercury vapor lamps (Sankyo denki, Japan) with the maximum light intensity of 254 nm and 365 nm. Photocatalytic experiments were carried out with a known amount (5 mg) of prepared nanophotocatalyst suspended in 50 ml of Cr(VI) and BPA aqueous solution with the concentration of 10 mg L^−1^ under stirring. Before starting the photodegradation experiments, the mixtures were stirred under dark for 30 min, to obtain the adsorption-desorption equilibrium. Five different parameters were studied in this study, viz. photocatalyst dosage (0.1 and 0.2 g L^−1^), varying pH (2 and 5.6), initial pollutant concentration (10, 25 and 50 mg L^−1^), lamp intensity (16 and 32 W) and wavelengths (254 and 365 nm). Each parameter was studied by keeping other parameters constant. At certain time intervals, 3 ml solution was taken out from the reactor and filtered using 0.45 µm filter. The filtrate was then analyzed by using UV-Visible spectrophotometer at 200–800 nm wavelength. The concentration of Cr(VI) was measured by spectrophotometric method using 1,5-diphenylcarazide as color reagent at 540 nm. The concentration of BPA was measured at 276 nm.

### Photoelectrochemical measurements

The photoelectrochemical properties of as prepared photocatalysts were investigated using CHI660C electrochemical workstation with a conventional three electrode system. Ag/AgCl, Pt-wire were served as a reference and counter electrodes and 0.1 M Na_2_SO_4_ aqueous solution was used as an electrolyte. The working electrode was prepared by the following procedure: 5 mg of photocatalyst was ground with 20 µL of deionized water and 10 µL of Triton X-100 to prepare slurry. Then the slurry was coated on the conductive side of fluorine doped tin oxide (FTO) plate with an active surface area of about 0.5 × 0.5 cm^2^ and then dried in hot air oven at 100 °C for about 6 h.

## Electronic supplementary material

Supplementary information

## Data Availability

All data generated or analyzed during this study are included in this published article (and its Supplementary Information files).
