# Supplementary material for: Photocatalytic degradation of toxic aquatic pollutants by novel magnetic 3D-TiO2@HPGA nanocomposite
Source: Sci Rep. 2018 Oct 19;8:15531. doi: 10.1038/s41598-018-33818-9 (PMC6195541; doi:10.1038/s41598-018-33818-9)
Supplement: Supplementary file 1 — Supplementary information [file 41598_2018_33818_MOESM1_ESM.pdf]

Supplementary Information

Photocatalytic degradation of toxic aquatic pollutants by novel magnetic 3D-TiO_2@HPGA nanocomposite

Chella Santhosh [a,b*], A. Malathi [c], Ehsan Daneshvar [a], Pratap Kollu [d,e,*] Amit Bhatnagar [a,*]

[a] *Department of Environmental and Biological Sciences, University of Eastern Finland, P.O. Box 1627, FI-70211 Kuopio, Finland*

[b] *Department of Electronics and Communication Engineering, KLEF, Greenfields, Vaddeswaram, Vijayawada 522502, India*

[c] *Solar Energy Lab, Department of Chemistry, Thiruvalluvar University, Vellore, 632115, India*

[d] *CASEST, School of Physics, University of Hyderabad, Gachibowli, Hyderabad 500046, India*

[e] *Newton Alumnus fellow, Thin film magnetism group, Cavendish Laboratory, University of Cambridge, Cambridge CB3 0HE, UK*

*Corresponding authors: raurisanthosh@gmail.com; pratapk@uohyd.ac.in; amit.bhatnagar@uef.fi

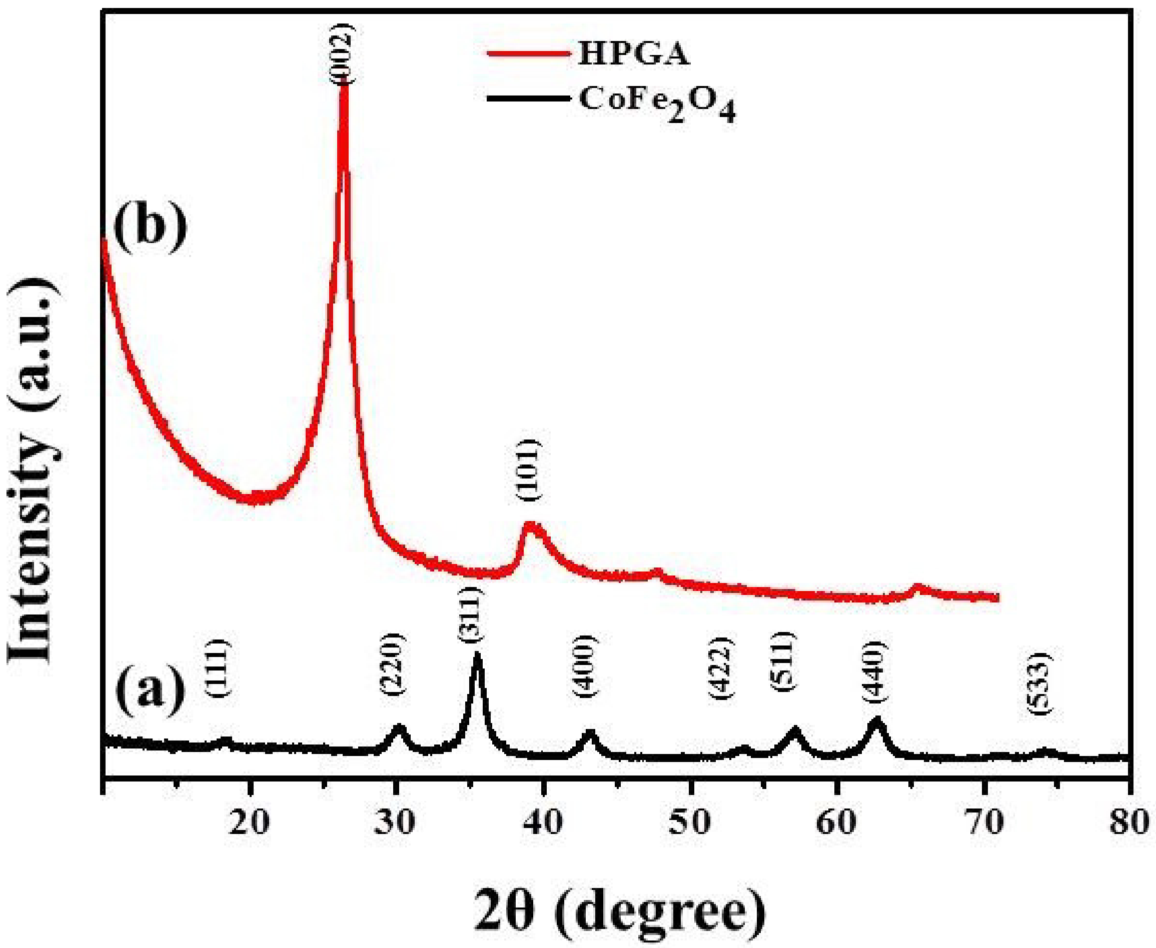

Figure S1. XRD analysis of as-prepared (a) bare $CoFe_2O_4$ and (b) HPGA nanomaterials.

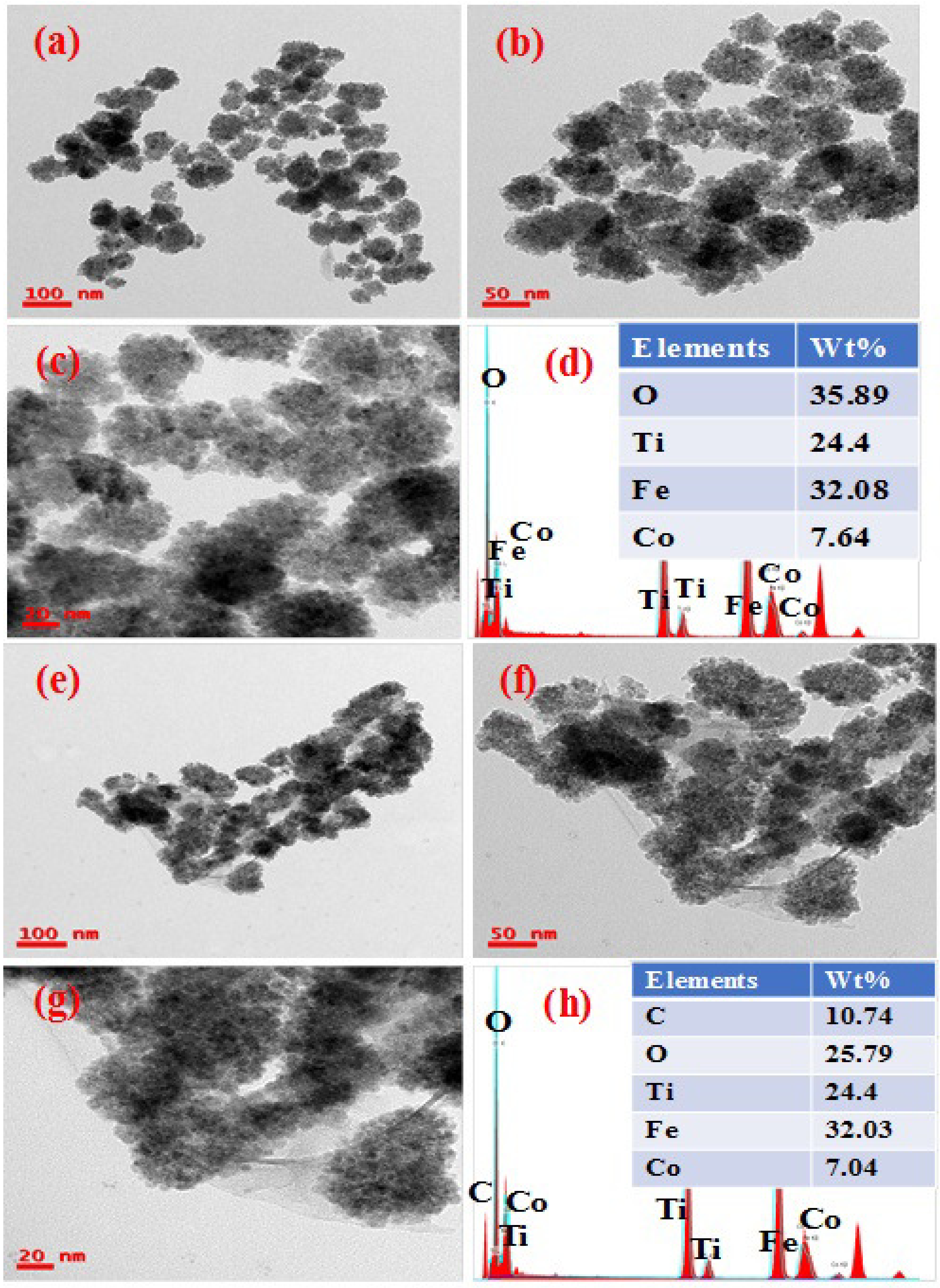

Figure S2. TEM images of (a-d) magnetic 3D-TiO_2 and (e-h) magnetic 3D-TiO_2@ HPGA with EDAX analysis.

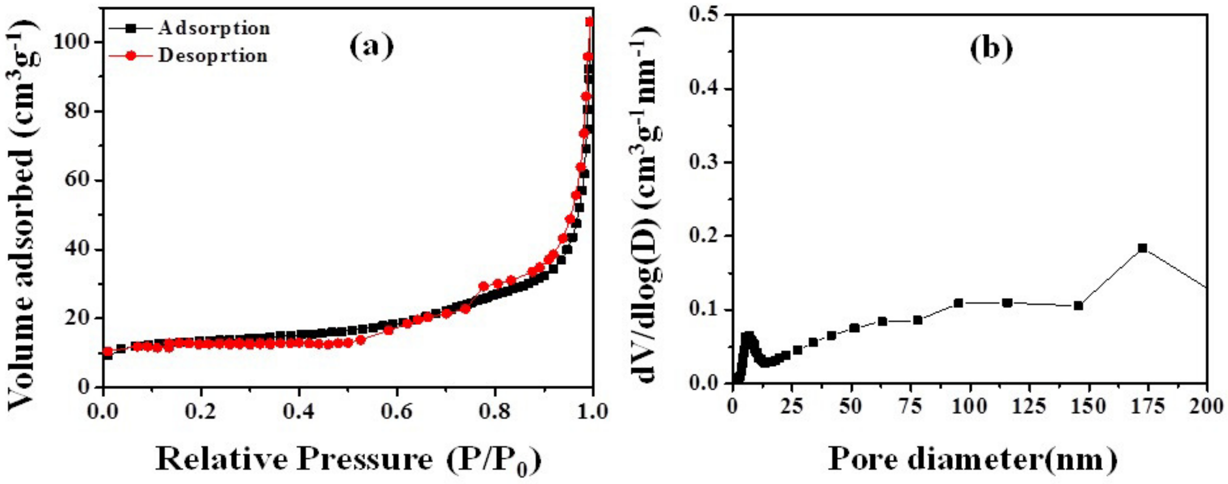

Figure S3. (a) N_2 adsorption/desorption isotherms and (b) BJH pore size distribution of prepared m3D-T-HPGA NC material.

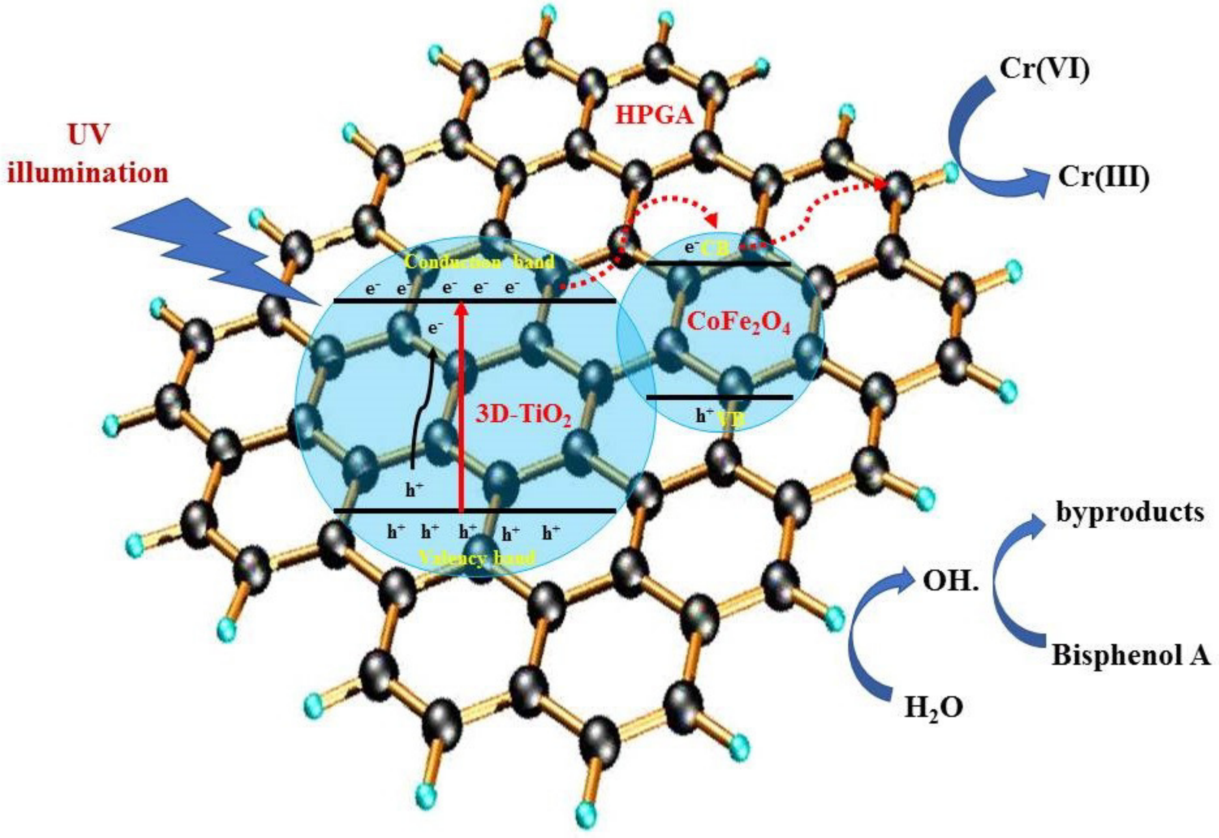

Figure S4. Proposed mechanism of photocatalytic degradation of Cr(VI) and BPA by the prepared nanocomposites.

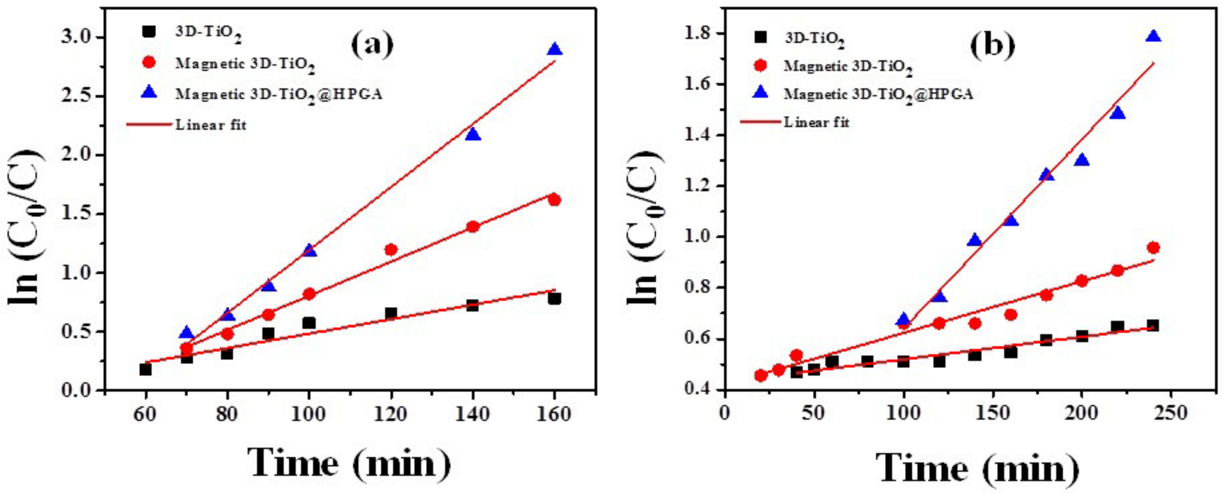

Figure S5. Pseudo first order model of (a) Cr(VI) and (b) BPA degradation under UV irradiation over 3D-TiO_2, magnetic 3D-TiO_2 and magnetic 3D-TiO_2@HPGA nanocomposites.

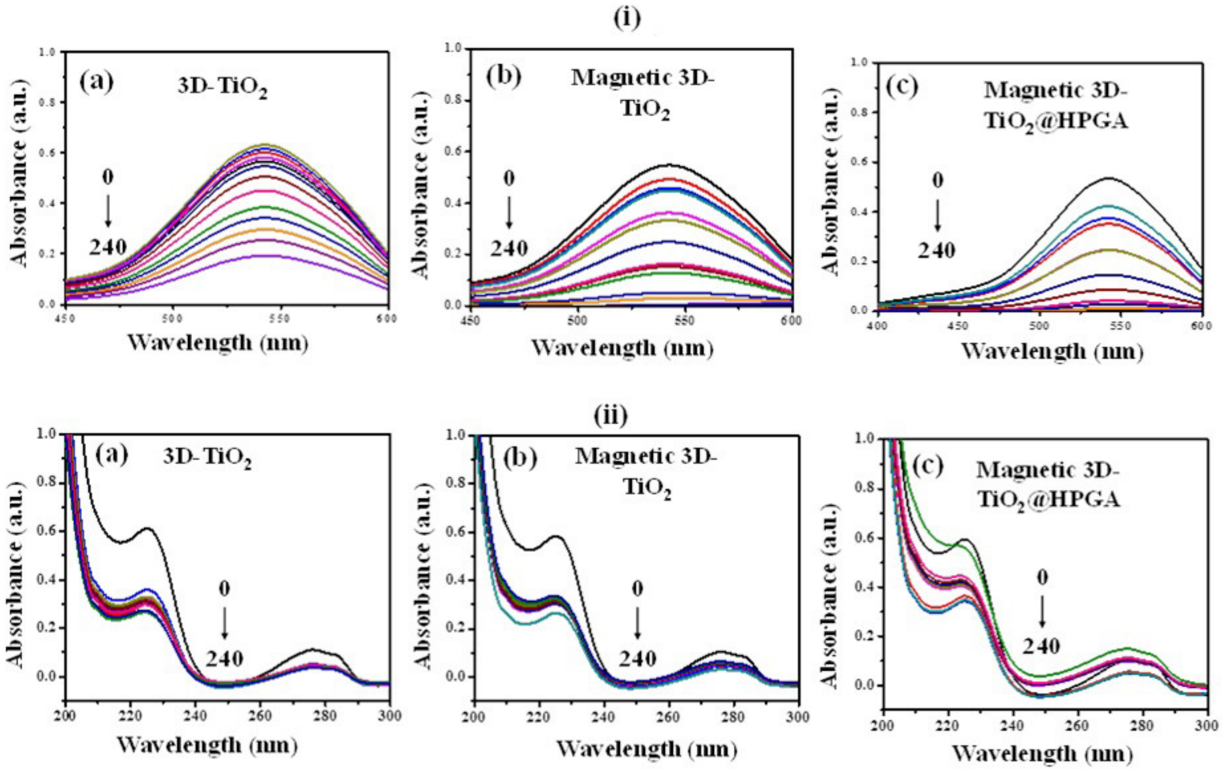

Figure S6. Absorbance spectra of (i) Cr(VI) and (ii) BPA as a function of irradiation time in the presence of prepared photocatalysts.
